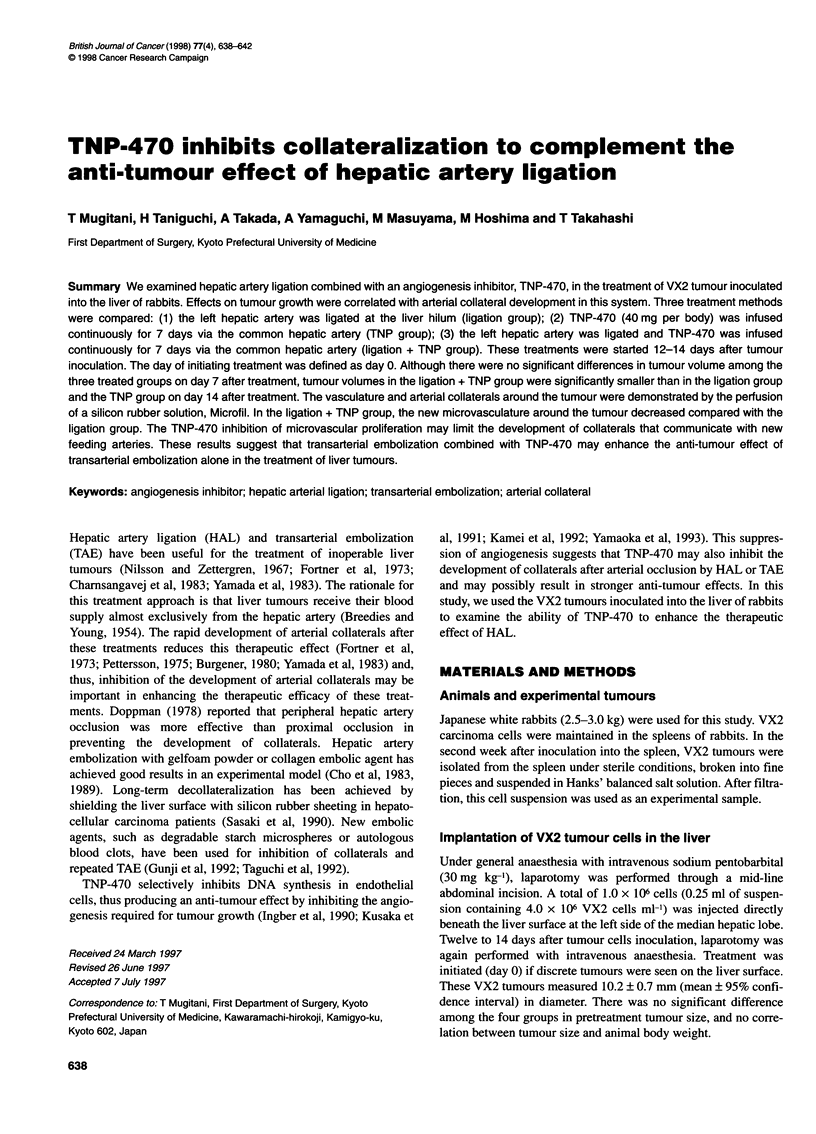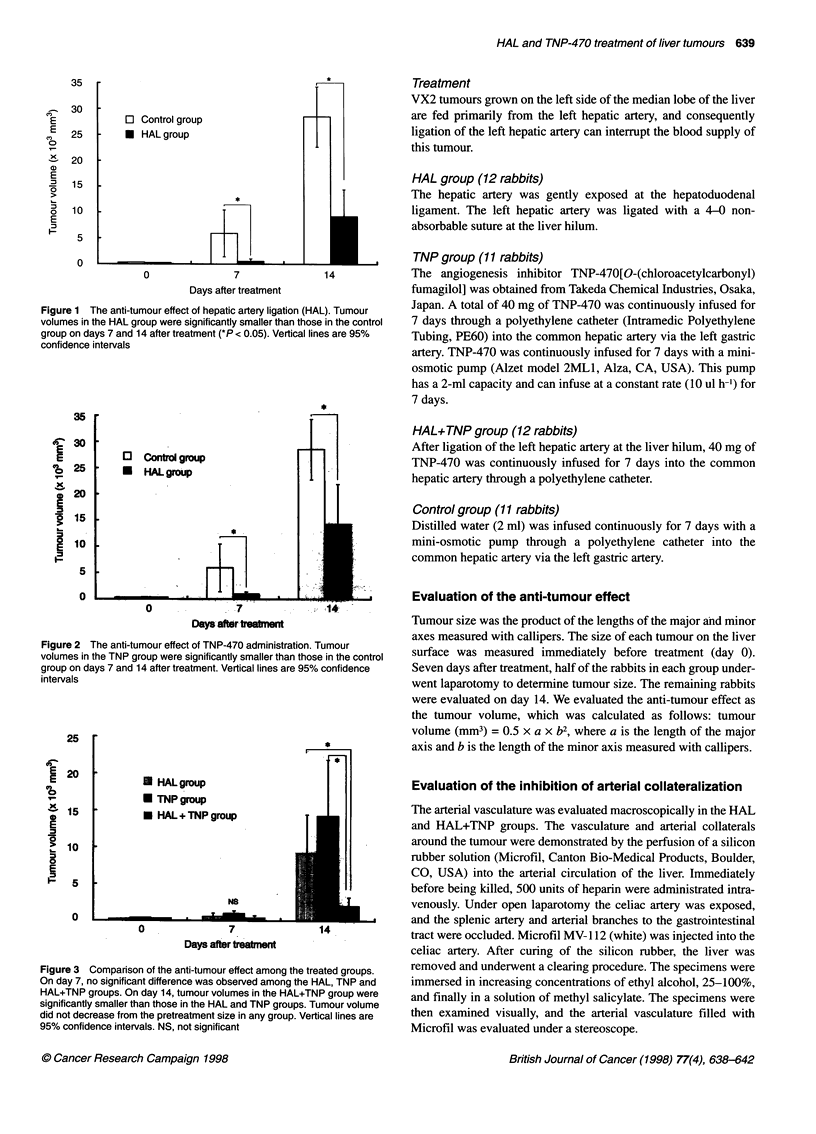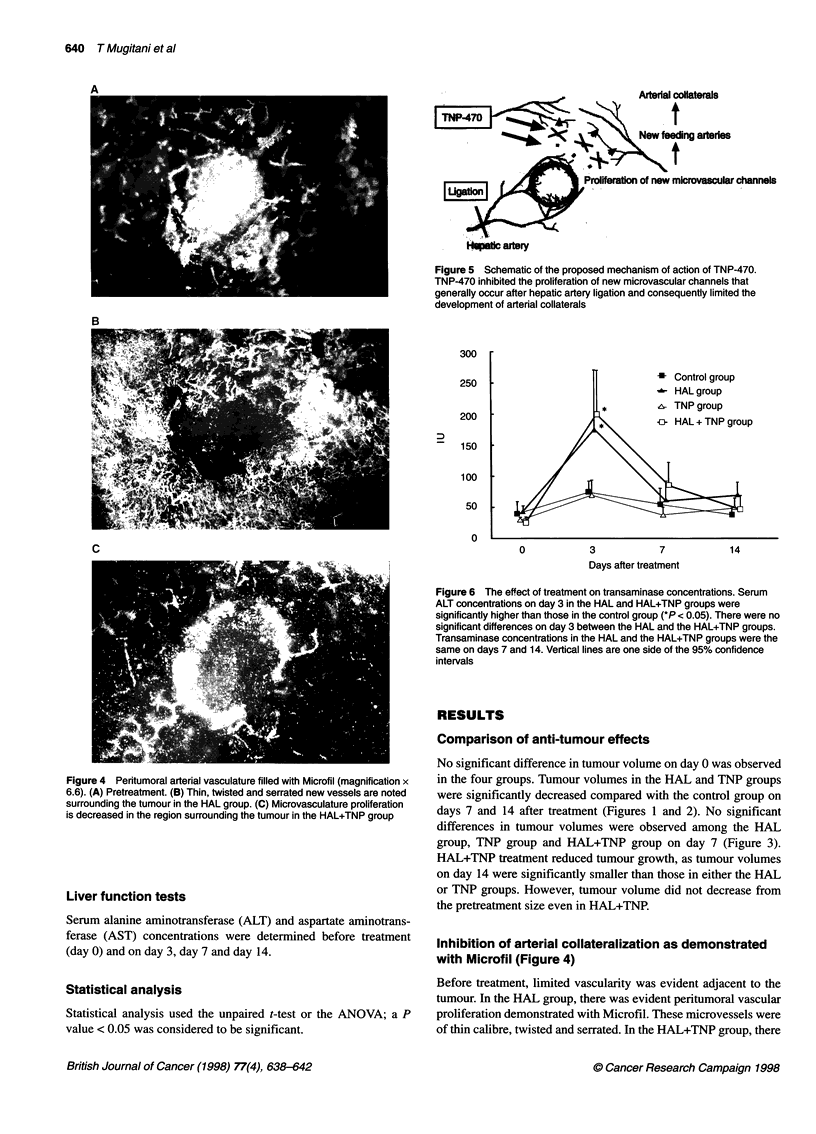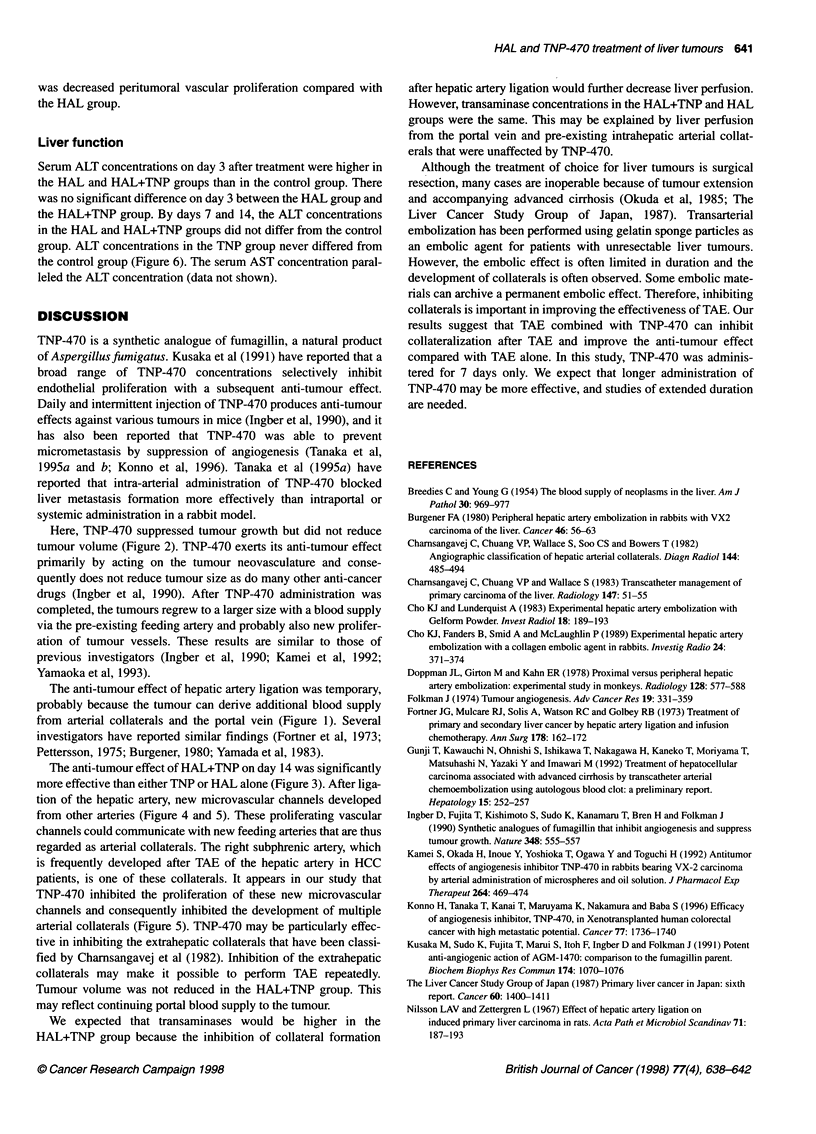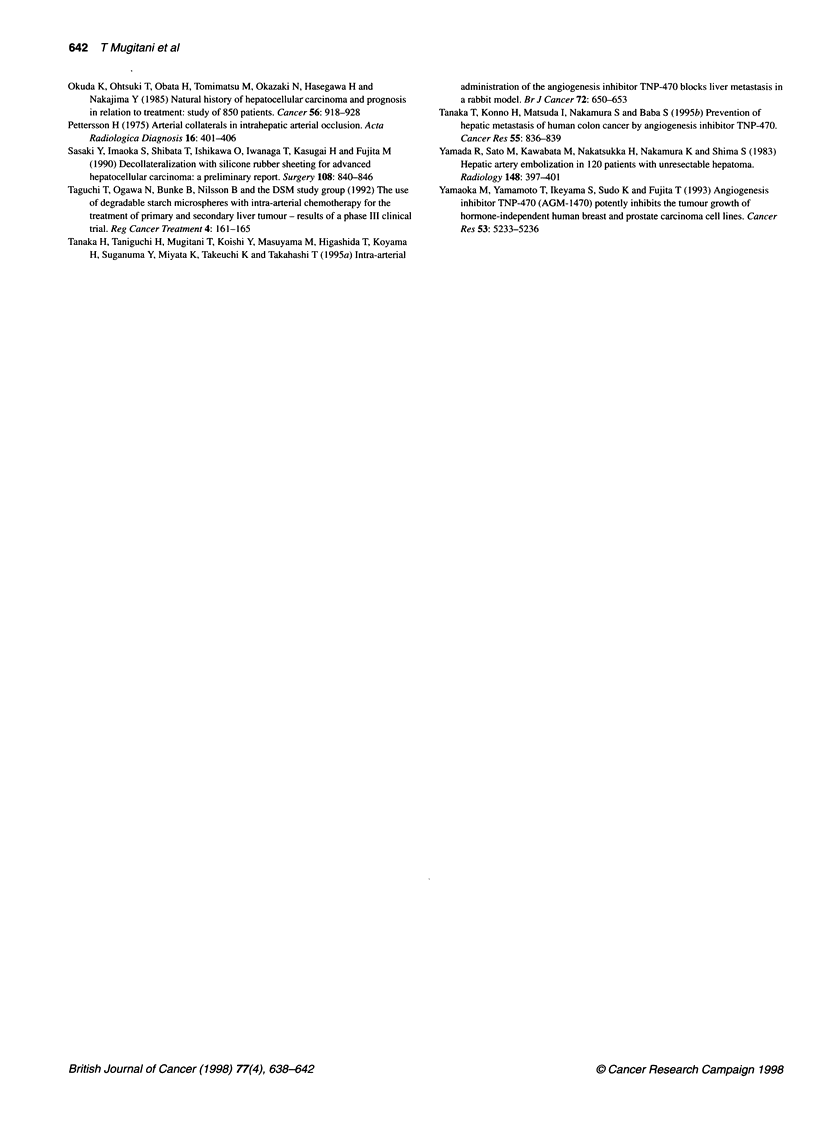# TNP-470 inhibits collateralization to complement the anti-tumour effect of hepatic artery ligation.

**DOI:** 10.1038/bjc.1998.102

**Published:** 1998-02

**Authors:** T. Mugitani, H. Taniguchi, A. Takada, A. Yamaguchi, M. Masuyama, M. Hoshima, T. Takahashi

**Affiliations:** First Department of Surgery, Kyoto Prefectural University of Medicine, Japan.

## Abstract

**Images:**


*British Journal of Cancer* (1998) 77(4), 638–642
© 1998 Cancer Research Campaign

### TNP-470 inhibits collateralization to complement the anti-tumour effect of hepatic artery ligation

**T Mugitani, H Taniguchi, A Takada, A Yamaguchi, M Masuyama, M Hoshima and T Takahashi**

First Department of Surgery, Kyoto Prefectural University of Medicine

**Summary** We examined hepatic artery ligation combined with an angiogenesis inhibitor, TNP-470, in the treatment of VX2 tumour inoculated into the liver of rabbits. Effects on tumour growth were correlated with arterial collateral development in this system. Three treatment methods were compared: (1) the left hepatic artery was ligated at the liver hilum (ligation group); (2) TNP-470 (40 mg per body) was infused continuously for 7 days via the common hepatic artery (TNP group); (3) the left hepatic artery was ligated and TNP-470 was infused continuously for 7 days via the common hepatic artery (ligation + TNP group). These treatments were started 12–14 days after tumour inoculation. The day of initiating treatment was defined as day 0. Although there were no significant differences in tumour volume among the three treated groups on day 7 after treatment, tumour volumes in the ligation + TNP group were significantly smaller than in the ligation group and the TNP group on day 14 after treatment. The vasculature and arterial collaterals around the tumour were demonstrated by the perfusion of a silicon rubber solution, Microfil. In the ligation + TNP group, the new microvasculature around the tumour decreased compared with the ligation group. The TNP-470 inhibition of microvascular proliferation may limit the development of collaterals that communicate with new feeding arteries. These results suggest that transarterial embolization combined with TNP-470 may enhance the anti-tumour effect of transarterial embolization alone in the treatment of liver tumours.

**Keywords:** angiogenesis inhibitor; hepatic arterial ligation; transarterial embolization; arterial collateral

Hepatic artery ligation (HAL) and transarterial embolization (TAE) have been useful for the treatment of inoperable liver tumours (Nilsson and Zettergren, 1967; Fortner et al, 1973; Charnsangavej et al, 1983; Yamada et al, 1983). The rationale for this treatment approach is that liver tumours receive their blood supply almost exclusively from the hepatic artery (Breedies and Young, 1954). The rapid development of arterial collaterals after these treatments reduces this therapeutic effect (Fortner et al, 1973; Pettersson, 1975; Burgener, 1980; Yamada et al, 1983) and, thus, inhibition of the development of arterial collaterals may be important in enhancing the therapeutic efficacy of these treatments. Doppman (1978) reported that peripheral hepatic artery occlusion was more effective than proximal occlusion in preventing the development of collaterals. Hepatic artery embolization with gelfoam powder or collagen embolic agent has achieved good results in an experimental model (Cho et al, 1983, 1989). Long-term decollateralization has been achieved by shielding the liver surface with silicon rubber sheeting in hepatocellular carcinoma patients (Sasaki et al, 1990). New embolic agents, such as degradable starch microspheres or autologous blood clots, have been used for inhibition of collaterals and repeated TAE (Gunji et al, 1992; Taguchi et al, 1992).

TNP-470 selectively inhibits DNA synthesis in endothelial cells, thus producing an anti-tumour effect by inhibiting the angiogenesis required for tumour growth (Ingber et al, 1990; Kusaka et al, 1991; Kamei et al, 1992; Yamaoka et al, 1993). This suppression of angiogenesis suggests that TNP-470 may also inhibit the development of collaterals after arterial occlusion by HAL or TAE and may possibly result in stronger anti-tumour effects. In this study, we used the VX2 tumours inoculated into the liver of rabbits to examine the ability of TNP-470 to enhance the therapeutic effect of HAL.

*Received 24 March 1997*
*Revised 26 June 1997*
*Accepted 7 July 1997*

*Correspondence to:* T Mugitani, First Department of Surgery, Kyoto Prefectural University of Medicine, Kawaramachi-hirokoji, Kamigyo-ku, Kyoto 602, Japan

### MATERIALS AND METHODS

### Animals and experimental tumours

Japanese white rabbits (2.5–3.0 kg) were used for this study. VX2 carcinoma cells were maintained in the spleens of rabbits. In the second week after inoculation into the spleen, VX2 tumours were isolated from the spleen under sterile conditions, broken into fine pieces and suspended in Hanks' balanced salt solution. After filtration, this cell suspension was used as an experimental sample.

### Implantation of VX2 tumour cells in the liver

Under general anaesthesia with intravenous sodium pentobarbital (30 mg $kg^{-1}$), laparotomy was performed through a mid-line abdominal incision. A total of $1.0 \times 10^6$ cells (0.25 ml of suspension containing $4.0 \times 10^6$ VX2 cells $ml^{-1}$) was injected directly beneath the liver surface at the left side of the median hepatic lobe. Twelve to 14 days after tumour cells inoculation, laparotomy was again performed with intravenous anaesthesia. Treatment was initiated (day 0) if discrete tumours were seen on the liver surface. These VX2 tumours measured $10.2 \pm 0.7$ mm (mean ± 95% confidence interval) in diameter. There was no significant difference among the four groups in pretreatment tumour size, and no correlation between tumour size and animal body weight.

**Figure 1** The anti-tumour effect of hepatic artery ligation (HAL). Tumour volumes in the HAL group were significantly smaller than those in the control group on days 7 and 14 after treatment (*$P < 0.05$). Vertical lines are 95% confidence intervals

**Figure 2** The anti-tumour effect of TNP-470 administration. Tumour volumes in the TNP group were significantly smaller than those in the control group on days 7 and 14 after treatment. Vertical lines are 95% confidence intervals

**Figure 3** Comparison of the anti-tumour effect among the treated groups. On day 7, no significant difference was observed among the HAL, TNP and HAL+TNP groups. On day 14, tumour volumes in the HAL+TNP group were significantly smaller than those in the HAL and TNP groups. Tumour volume did not decrease from the pretreatment size in any group. Vertical lines are 95% confidence intervals. NS, not significant

*Treatment*

VX2 tumours grown on the left side of the median lobe of the liver are fed primarily from the left hepatic artery, and consequently ligation of the left hepatic artery can interrupt the blood supply of this tumour.

*HAL group (12 rabbits)*

The hepatic artery was gently exposed at the hepatoduodenal ligament. The left hepatic artery was ligated with a 4–0 non-absorbable suture at the liver hilum.

*TNP group (11 rabbits)*

The angiogenesis inhibitor TNP-470[*O*-(chloroacetylcarbonyl) fumagilol] was obtained from Takeda Chemical Industries, Osaka, Japan. A total of 40 mg of TNP-470 was continuously infused for 7 days through a polyethylene catheter (Intramedic Polyethylene Tubing, PE60) into the common hepatic artery via the left gastric artery. TNP-470 was continuously infused for 7 days with a mini-osmotic pump (Alzet model 2ML1, Alza, CA, USA). This pump has a 2-ml capacity and can infuse at a constant rate (10 ul $h^{-1}$) for 7 days.

*HAL+TNP group (12 rabbits)*

After ligation of the left hepatic artery at the liver hilum, 40 mg of TNP-470 was continuously infused for 7 days into the common hepatic artery through a polyethylene catheter.

*Control group (11 rabbits)*

Distilled water (2 ml) was infused continuously for 7 days with a mini-osmotic pump through a polyethylene catheter into the common hepatic artery via the left gastric artery.

### Evaluation of the anti-tumour effect

Tumour size was the product of the lengths of the major and minor axes measured with callipers. The size of each tumour on the liver surface was measured immediately before treatment (day 0). Seven days after treatment, half of the rabbits in each group underwent laparotomy to determine tumour size. The remaining rabbits were evaluated on day 14. We evaluated the anti-tumour effect as the tumour volume, which was calculated as follows: tumour volume ($mm^3$) = $0.5 \times a \times b^2$, where $a$ is the length of the major axis and $b$ is the length of the minor axis measured with callipers.

### Evaluation of the inhibition of arterial collateralization

The arterial vasculature was evaluated macroscopically in the HAL and HAL+TNP groups. The vasculature and arterial collaterals around the tumour were demonstrated by the perfusion of a silicon rubber solution (Microfil, Canton Bio-Medical Products, Boulder, CO, USA) into the arterial circulation of the liver. Immediately before being killed, 500 units of heparin were administrated intravenously. Under open laparotomy the celiac artery was exposed, and the splenic artery and arterial branches to the gastrointestinal tract were occluded. Microfil MV-112 (white) was injected into the celiac artery. After curing of the silicon rubber, the liver was removed and underwent a clearing procedure. The specimens were immersed in increasing concentrations of ethyl alcohol, 25–100%, and finally in a solution of methyl salicylate. The specimens were then examined visually, and the arterial vasculature filled with Microfil was evaluated under a stereoscope.

© Cancer Research Campaign 1998

Figure 4 Peritumoral arterial vasculature filled with Microfil (magnification × 6.6). (A) Pretreatment. (B) Thin, twisted and serrated new vessels are noted surrounding the tumour in the HAL group. (C) Microvasculature proliferation is decreased in the region surrounding the tumour in the HAL+TNP group

Figure 5 Schematic of the proposed mechanism of action of TNP-470. TNP-470 inhibited the proliferation of new microvascular channels that generally occur after hepatic artery ligation and consequently limited the development of arterial collaterals

Figure 6 The effect of treatment on transaminase concentrations. Serum ALT concentrations on day 3 in the HAL and HAL+TNP groups were significantly higher than those in the control group (*$P < 0.05$). There were no significant differences on day 3 between the HAL and the HAL+TNP groups. Transaminase concentrations in the HAL and the HAL+TNP groups were the same on days 7 and 14. Vertical lines are one side of the 95% confidence intervals

### Liver function tests

Serum alanine aminotransferase (ALT) and aspartate aminotransferase (AST) concentrations were determined before treatment (day 0) and on day 3, day 7 and day 14.

### Statistical analysis

Statistical analysis used the unpaired *t*-test or the ANOVA; a $P$ value < 0.05 was considered to be significant.

### RESULTS

### Comparison of anti-tumour effects

No significant difference in tumour volume on day 0 was observed in the four groups. Tumour volumes in the HAL and TNP groups were significantly decreased compared with the control group on days 7 and 14 after treatment (Figures 1 and 2). No significant differences in tumour volumes were observed among the HAL group, TNP group and HAL+TNP group on day 7 (Figure 3). HAL+TNP treatment reduced tumour growth, as tumour volumes on day 14 were significantly smaller than those in either the HAL or TNP groups. However, tumour volume did not decrease from the pretreatment size even in HAL+TNP.

### Inhibition of arterial collateralization as demonstrated with Microfil (Figure 4)

Before treatment, limited vascularity was evident adjacent to the tumour. In the HAL group, there was evident peritumoral vascular proliferation demonstrated with Microfil. These microvessels were of thin calibre, twisted and serrated. In the HAL+TNP group, there

© Cancer Research Campaign 1998

was decreased peritumoral vascular proliferation compared with the HAL group.

### Liver function

Serum ALT concentrations on day 3 after treatment were higher in the HAL and HAL+TNP groups than in the control group. There was no significant difference on day 3 between the HAL group and the HAL+TNP group. By days 7 and 14, the ALT concentrations in the HAL and HAL+TNP groups did not differ from the control group. ALT concentrations in the TNP group never differed from the control group (Figure 6). The serum AST concentration paralleled the ALT concentration (data not shown).

### DISCUSSION

TNP-470 is a synthetic analogue of fumagillin, a natural product of *Aspergillus fumigatus*. Kusaka et al (1991) have reported that a broad range of TNP-470 concentrations selectively inhibit endothelial proliferation with a subsequent anti-tumour effect. Daily and intermittent injection of TNP-470 produces anti-tumour effects against various tumours in mice (Ingber et al, 1990), and it has also been reported that TNP-470 was able to prevent micrometastasis by suppression of angiogenesis (Tanaka et al, 1995*a* and *b*; Konno et al, 1996). Tanaka et al (1995*a*) have reported that intra-arterial administration of TNP-470 blocked liver metastasis formation more effectively than intraportal or systemic administration in a rabbit model.

Here, TNP-470 suppressed tumour growth but did not reduce tumour volume (Figure 2). TNP-470 exerts its anti-tumour effect primarily by acting on the tumour neovasculature and consequently does not reduce tumour size as do many other anti-cancer drugs (Ingber et al, 1990). After TNP-470 administration was completed, the tumours regrew to a larger size with a blood supply via the pre-existing feeding artery and probably also new proliferation of tumour vessels. These results are similar to those of previous investigators (Ingber et al, 1990; Kamei et al, 1992; Yamaoka et al, 1993).

The anti-tumour effect of hepatic artery ligation was temporary, probably because the tumour can derive additional blood supply from arterial collaterals and the portal vein (Figure 1). Several investigators have reported similar findings (Fortner et al, 1973; Pettersson, 1975; Burgener, 1980; Yamada et al, 1983).

The anti-tumour effect of HAL+TNP on day 14 was significantly more effective than either TNP or HAL alone (Figure 3). After ligation of the hepatic artery, new microvascular channels developed from other arteries (Figure 4 and 5). These proliferating vascular channels could communicate with new feeding arteries that are thus regarded as arterial collaterals. The right subphrenic artery, which is frequently developed after TAE of the hepatic artery in HCC patients, is one of these collaterals. It appears in our study that TNP-470 inhibited the proliferation of these new microvascular channels and consequently inhibited the development of multiple arterial collaterals (Figure 5). TNP-470 may be particularly effective in inhibiting the extrahepatic collaterals that have been classified by Charnsangavej et al (1982). Inhibition of the extrahepatic collaterals may make it possible to perform TAE repeatedly. Tumour volume was not reduced in the HAL+TNP group. This may reflect continuing portal blood supply to the tumour.

We expected that transaminases would be higher in the HAL+TNP group because the inhibition of collateral formation after hepatic artery ligation would further decrease liver perfusion. However, transaminase concentrations in the HAL+TNP and HAL groups were the same. This may be explained by liver perfusion from the portal vein and pre-existing intrahepatic arterial collaterals that were unaffected by TNP-470.

Although the treatment of choice for liver tumours is surgical resection, many cases are inoperable because of tumour extension and accompanying advanced cirrhosis (Okuda et al, 1985; The Liver Cancer Study Group of Japan, 1987). Transarterial embolization has been performed using gelatin sponge particles as an embolic agent for patients with unresectable liver tumours. However, the embolic effect is often limited in duration and the development of collaterals is often observed. Some embolic materials can archive a permanent embolic effect. Therefore, inhibiting collaterals is important in improving the effectiveness of TAE. Our results suggest that TAE combined with TNP-470 can inhibit collateralization after TAE and improve the anti-tumour effect compared with TAE alone. In this study, TNP-470 was administered for 7 days only. We expect that longer administration of TNP-470 may be more effective, and studies of extended duration are needed.

### REFERENCES

Breedies C and Young G (1954) The blood supply of neoplasms in the liver. *Am J Pathol* **30**: 969–977

Burgener FA (1980) Peripheral hepatic artery embolization in rabbits with VX2 carcinoma of the liver. *Cancer* **46**: 56–63

Charnsangavej C, Chuang VP, Wallace S, Soo CS and Bowers T (1982) Angiographic classification of hepatic arterial collaterals. *Diagn Radiol* **144**: 485–494

Charnsangavej C, Chuang VP and Wallace S (1983) Transcatheter management of primary carcinoma of the liver. *Radiology* **147**: 51–55

Cho KJ and Lunderquist A (1983) Experimental hepatic artery embolization with Gelform Powder. *Invest Radiol* **18**: 189–193

Cho KJ, Fanders B, Smid A and McLaughlin P (1989) Experimental hepatic artery embolization with a collagen embolic agent in rabbits. *Investig Radio* **24**: 371–374

Doppman JL, Girton M and Kahn ER (1978) Proximal versus peripheral hepatic artery embolization: experimental study in monkeys. *Radiology* **128**: 577–588

Folkman J (1974) Tumour angiogenesis. *Adv Cancer Res* **19**: 331–359

Fortner JG, Mulcare RJ, Solis A, Watson RC and Golbey RB (1973) Treatment of primary and secondary liver cancer by hepatic artery ligation and infusion chemotherapy. *Ann Surg* **178**: 162–172

Gunji T, Kawauchi N, Ohnishi S, Ishikawa T, Nakagawa H, Kaneko T, Moriyama T, Matsuhashi N, Yazaki Y and Imawari M (1992) Treatment of hepatocellular carcinoma associated with advanced cirrhosis by transcatheter arterial chemoembolization using autologous blood clot: a preliminary report. *Hepatology* **15**: 252–257

Ingber D, Fujita T, Kishimoto S, Sudo K, Kanamaru T, Bren H and Folkman J (1990) Synthetic analogues of fumagillin that inhibit angiogenesis and suppress tumour growth. *Nature* **348**: 555–557

Kamei S, Okada H, Inoue Y, Yoshioka T, Ogawa Y and Toguchi H (1992) Antitumor effects of angiogenesis inhibitor TNP-470 in rabbits bearing VX-2 carcinoma by arterial administration of microspheres and oil solution. *J Pharmacol Exp Therapeut* **264**: 469–474

Konno H, Tanaka T, Kanai T, Maruyama K, Nakamura and Baba S (1996) Efficacy of angiogenesis inhibitor, TNP-470, in Xenotransplanted human colorectal cancer with high metastatic potential. *Cancer* **77**: 1736–1740

Kusaka M, Sudo K, Fujita T, Marui S, Itoh F, Ingber D and Folkman J (1991) Potent anti-angiogenic action of AGM-1470: comparison to the fumagillin parent. *Biochem Biophys Res Commun* **174**: 1070–1076

The Liver Cancer Study Group of Japan (1987) Primary liver cancer in Japan: sixth report. *Cancer* **60**: 1400–1411

Nilsson LAV and Zettergren L (1967) Effect of hepatic artery ligation on induced primary liver carcinoma in rats. *Acta Path et Microbiol Scandinav* **71**: 187–193

© Cancer Research Campaign 1998

Okuda K, Ohtsuki T, Obata H, Tomimatsu M, Okazaki N, Hasegawa H and Nakajima Y (1985) Natural history of hepatocellular carcinoma and prognosis in relation to treatment: study of 850 patients. *Cancer* **56**: 918–928

Pettersson H (1975) Arterial collaterals in intrahepatic arterial occlusion. *Acta Radiologica Diagnosis* **16**: 401–406

Sasaki Y, Imaoka S, Shibata T, Ishikawa O, Iwanaga T, Kasugai H and Fujita M (1990) Decollateralization with silicone rubber sheeting for advanced hepatocellular carcinoma: a preliminary report. *Surgery* **108**: 840–846

Taguchi T, Ogawa N, Bunke B, Nilsson B and the DSM study group (1992) The use of degradable starch microspheres with intra-arterial chemotherapy for the treatment of primary and secondary liver tumour – results of a phase III clinical trial. *Reg Cancer Treatment* **4**: 161–165

Tanaka H, Taniguchi H, Mugitani T, Koishi Y, Masuyama M, Higashida T, Koyama H, Suganuma Y, Miyata K, Takeuchi K and Takahashi T (1995*a*) Intra-arterial administration of the angiogenesis inhibitor TNP-470 blocks liver metastasis in a rabbit model. *Br J Cancer* **72**: 650–653

Tanaka T, Konno H, Matsuda I, Nakamura S and Baba S (1995*b*) Prevention of hepatic metastasis of human colon cancer by angiogenesis inhibitor TNP-470. *Cancer Res* **55**: 836–839

Yamada R, Sato M, Kawabata M, Nakatsukka H, Nakamura K and Shima S (1983) Hepatic artery embolization in 120 patients with unresectable hepatoma. *Radiology* **148**: 397–401

Yamaoka M, Yamamoto T, Ikeyama S, Sudo K and Fujita T (1993) Angiogenesis inhibitor TNP-470 (AGM-1470) potently inhibits the tumour growth of hormone-independent human breast and prostate carcinoma cell lines. *Cancer Res* **53**: 5233–5236

© Cancer Research Campaign 1998